# Multimodal ultrasonography findings of extramammary granular cell tumors: Two case reports

**DOI:** 10.3389/fonc.2023.1136770

**Published:** 2023-03-20

**Authors:** Meng Zhu, Huan Xu, Yujuan Chen, Yulan Peng

**Affiliations:** ^1^ Department of Ultrasound, West China Hospital, Sichuan University, Chengdu, China; ^2^ Department of Pathology, West China Hospital, Sichuan University, Chengdu, China; ^3^ Department of Breast Surgery, West China Hospital, Sichuan University, Chengdu, China

**Keywords:** granular cell tumor, contrast-enhanced ultrasound, shear wave elasticity, pectoralis major muscle, axillary, case reports

## Abstract

Extramammary masses are infrequently encountered in breast examinations. They may occur in the chest wall and axilla as neighbors of the breast. It is important to determine the nature of the lesion. However, some benign tumors, such as granular cell tumors (GCTs), also show malignant characteristics, which leads to misdiagnosis. To the best of our knowledge, multimodal ultrasound features of GCT have not been elucidated. We report two cases of women with GCTs encountered upon breast cancer screening; the tumor was not located in breast tissue. The first patient was a 37-year-old woman who presented with a slow-growing mass in the right breast and the GCT was located in the pectoralis major muscle. The second patient was a 52-year-old woman who presented with a palpable left axillary mass and the GCT was located in the axilla. Mammography failed to detect the masses in the two patients upon breast cancer screening. However, two-dimensional ultrasonography revealed a solid heterogeneous hypoechoic mass. Shear wave elastography showed that the masses had an increased hardness compared with the surrounding tissue. Further contrast-enhanced ultrasonography showed that the contrast patterns of the two masses were different. In case one, contrast-enhanced ultrasonography showed an inhomogeneous annular high enhancement, and the dynamic curve showed rapid enhancement and regression. In case two, contrast enhanced ultrasound showed slight enhancement around the lesion but no enhancement inside. Postoperative pathology confirmed that the GCT was benign in both cases. The patients showed no signs of recurrence at the 2-year follow-up. Here, we report two cases and present the multimodal ultrasonography findings of this tumor for the first time. Radiologists and surgeons should be aware of these imaging manifestations and include them in their differential diagnoses.

## 1 Introduction

Granular cell tumor (GCT) is a rare type of tumor originating from the Schwann cells of peripheral nerves and is usually encountered in the head, neck, and tongue regions (1, 2). GCT is composed of cell clusters and niduses of eosinophilic cytoplasmic granules with vesicular nuclei. The presence of neuronal markers with diffuse S-100, CD68, and vimentin expression may be used to distinguish GCT from other granular lesions (3, 4). Histologically, cells in benign GCT commonly do not undergo mitosis and necrosis, and the Ki-67/MIB-1 proliferation index of benign GCT is usually less than 10% (3). Moreover, 2.5% of GCT cases are identified as malignant due to the presence of metastasis (5).

In clinical practice, for suspected breast masses, imaging examination is carried out to find out whether there is a breast lesion, and attention is paid to the condition of the axilla. Ultrasound examination of the breast and axilla is mainly used to evaluate symptomatic patients or further investigate findings determined by other imaging methods (6). However, ultrasound doctors may infrequently find an extramammary mass in the anterior chest wall or axilla when scanning the breast (6, 7). When that occurs, doctors should first determine the anatomical relationship between the mass and the breast, and then determine the nature of the mass to assess whether urgent treatment or referral is needed.

GCT is occasionally encountered in breast tissue or extramammary areas during breast ultrasound examination (8–11). Even GCT in breast parenchyma can cause extensive infiltration of the chest wall (12). When GCT occurs in extramammary locations, like the pectoral muscle, it manifests clinically as a painless solid mass mimicking a carcinoma (10). When it occurs in the axilla it may be mistaken as cancer; and when the axillary nerve is involved, it can cause limb pain (13). Several cases of GCT occurring in the anterior chest wall and axilla have been reported (9–15); however, details on the imaging findings in these cases are limited.

To the best of our knowledge, multimodal ultrasound imaging findings of GCT has not been reported to date. Herein, we report two cases of patients with GCT and focus primarily on their multimodal ultrasound imaging characteristics.

## 2 Case presentation

### 2.1 Case 1

**Figure 1:**
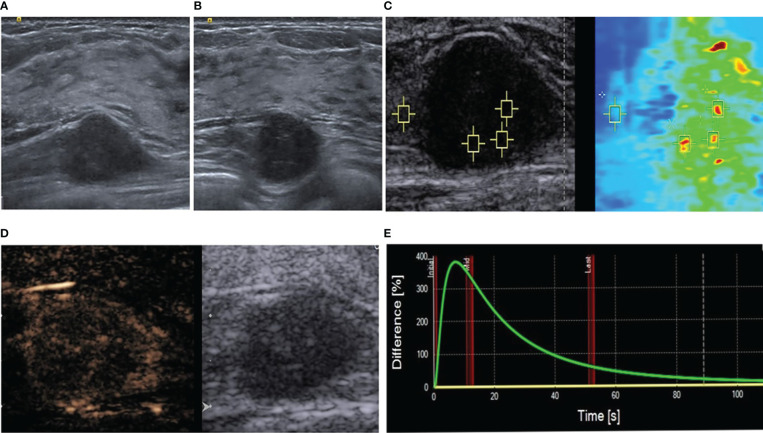
Multimodal ultrasonographic assessment of the mass. **(A)** Long-axis ultrasound showing a hypoechoic mass located deep in the pectoralis major muscle; it was deep in the gland and the upper right breast. **(B)** The adjacent glands appear blurred. **(C)** Virtual touch tissue imaging and quantification mode. The depth is 2.4 cm, median speed 6.06 m/s, and mean speed 6.18 m/s. The surrounding muscular layer is 2.72 m/s. **(D)** Qualitative contrast-enhanced ultrasound results. **(E)** Time-intensity curve.

A 37-year-old woman presented with a right-sided chest wall mass that had been gradually increasing in size for 8 months. The patient denied her cancer history. No previous surgical history was recorded. A physical examination revealed a solid mass at the outer upper quadrant in the right breast. No enlarged lymph nodes were palpable in the axilla. Notably, this mass was not apparent on mammography. Therefore, she underwent an ultrasonographic examination, which was performed using a 9-4-MHz linear array probe (Siemens, ACUSON Oxana 2 instrument). The ultrasonography revealed a solid hypoechoic mass that measured approximately 18 × 21 × 12 mm in the muscular layer of the chest wall (**Figure 1A**). The boundary of the mass was blurred (**Figure 1B**).

To further determine the nature of the mass, she underwent a multimodal ultrasonography. The examination was performed by a breast specialist with 15 years of experience. Shear wave elastography showed that the lesion was unevenly hard. Quantitative analysis using virtual touch quantification showed a mean shear wave speed of 6.18 m/s (**Figure 1C**). To evaluate the angiogenesis of the mass, the patient underwent a real-time contrast-enhanced ultrasonographic examination (CEUS). For the CEUS, 4.8 mL of the SonoVue (Bracco, Milan, Italy) contrast agent was administered by a bolus injection *via* the elbow vein and observed continuously for two minutes.

The qualitative CEUS results demonstrated an uneven high enhancement in the entire mass, annulus eccentricity enhancement, and a “dark star” sign (**Figure 1D**). The enhancement boundary was clear; the range before enhancement was 21x12 mm, and that after enhancement was 24x13 mm. The quantitative analysis of the CEUS images was conducted using the SonoLiver^®^ Software, which showed a rapid enhancement (**Figure 1E**). When the whole lesion was selected as the area of interest for analysis, the time taken to initiation of enhancement was 7.61 seconds, time to peak enhancement was 8.23 seconds, and the average transit time of contrast agent was 37.73 seconds.

Ultrasound-guided biopsy of the tumor was performed using a 16-G needle. The tumor cells had abundant cytoplasm, eosinophils, and sheet nest-like growth when visualized under a light microscope. Immunohistochemistry showed that the tumor cells were positive for S-100 and CD68 (PGM-1), and negative for pan-cytokeratin and neuron-specific enolase. The Ki-67/MIB-1 positivity rate was 2%. A diagnosis of GCT was made based on the results of pathological and immunohistochemical analyses.

**Figure 2:**
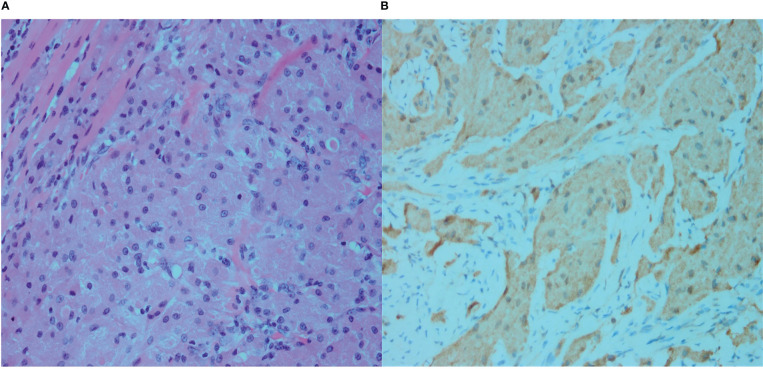
**(A)** Microscopy showing dense granular cells that are diffusely distributed, as well as infiltration and growth of the granular cells between the muscle fibers, with no clear separation between the cells and the muscle fibers (Hematoxylin and eosin [H&E] staining, ×400). **(B)** S-100. The cytoplasm of the tumor cells is brownish yellow, indicating strong positive staining (immunostaining, ×400).

The mass in the right pectoralis major muscle was completely resected. Postoperative pathology confirmed the presence of a GCT (**Figure 2**). The patient underwent annual ultrasound examinations, and no recurrence was noted during the 2-year follow-up.

### 2.2 Case 2

**Figure 3:**
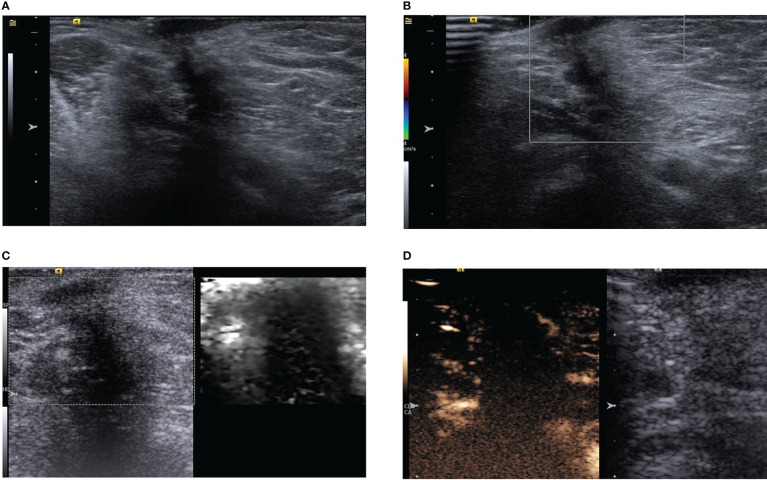
**(A)** Ultrasound showing a hypoechoic subcutaneous mass in the left axilla with an aspect ratio greater than one and acoustic shadowing. **(B)** Color Doppler shows no obvious blood supply in the mass. **(C)** Acoustic radiation force impulse model. **(D)** In the arterial phase, the tumor is slightly enhanced in the periphery, but not in the interior.

A 52-year-old woman had a palpable left axillary mass for a year before she visited our hospital. Although the patient denied any sensation of pain in the mass; abnormally red, swollen skin with discharge and progressive changes were observed. The patient had no relevant history of familial breast cancer or previous interventional procedures. A physical examination revealed a palpable, hard-textured mass of approximately 1.5×1.5 cm at the left axilla in proximity to the skin. The skin on the surface of the mass was ulcerated. No palpable right axillary or bilateral supraclavicular lymph nodes were found. The mass was not apparent on mammography. Subsequently, the patient underwent ultrasonography revealing a solid subcutaneous soft tissue mass of approximately 13×9×15 mm in the axilla, with unclear boundaries, irregular shape, and an aspect ratio greater than one (**Figure 3A**). Color Doppler ultrasonography demonstrated a lack of blood vessels in the mass (**Figure 3B**).

Multimodal ultrasonography was performed with the same ultrasound machine and settings used in case one. The ultrasound elastography revealed a hard-textured mass (**Figure 3C**) and contrast-enhanced ultrasonography indicated low enhancement in the peripheral part of the mass but no enhancement in the interior during the two minutes of continuous observation (**Figure 3D**). CEUS further revealed the possibility of potential benign lesions.

The patient underwent preoperative core needle biopsy. The results suggested the presence of cells rich in cytoplasmic granules growing as sheets or lumps in the fibrous connective tissue; upon immunohistochemical testing, tumor cells were strongly positive for S-100, weakly positive for CD68 (PGM-1), partially positive for transcription factor E3, and negative for CD163, cytokeratin, epithelial membrane antigen, human melanoma black-45 (HMB-45), chromogranin A, and thyroid transcription factor-1, which supported the diagnosis of a GCT.

The patient was subjected to preoperative magnetic resonance imaging (MRI) to further confirm the extent of the lesion. The T1- and T2-weighted scans revealed a slightly long nodular signal shadow of approximately 2.3×1.9 cm in the left axilla, with an irregular shape, rough edges, unclear boundaries in the deep surface of the muscle, and a slight thickening in proximity to the skin; the enhanced MRI showed an uneven enhancement.

**Figure 4:**
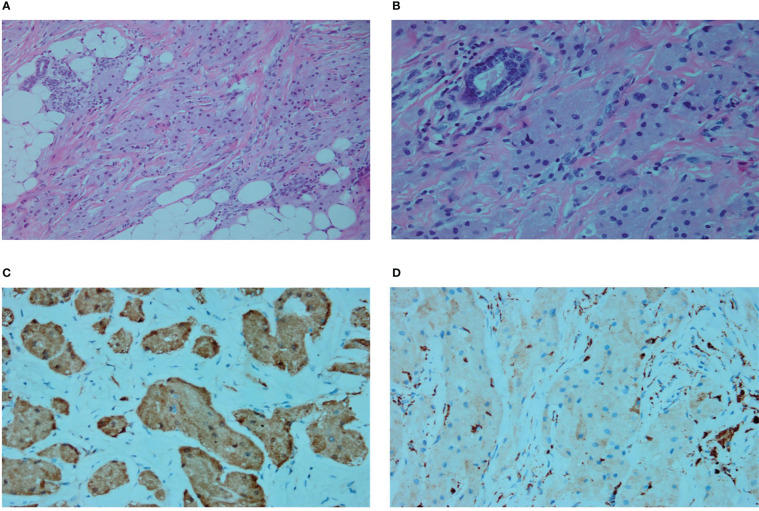
**(A)** Microscopic subcutaneous growth of infiltrative platelet-like growths of granulosa cells and vacuolated adipocytes are shown (Hematoxylin and eosin [H&E] staining, ×200). **(B)** In the fibrous septa, nest-like unencapsulated granulosa cells are also seen (H&E staining, ×400). **(C)** S100 cytoplasm showing diffuse and strong positivity (immunostaining, ×400). **(D)** CD68 weak positivity (immunostaining, ×400).

The patient underwent resection of the axillary mass and biopsy of the axillary lymph nodes under general anesthesia. The axillary tissues containing the mass were completely resected with a 3–5 cm resection range. In addition, four hard-textured lymph nodes of approximately 0.5–1.5 cm were found in the left axilla. While postoperative paraffin section examinations confirmed the diagnosis of GCT (**Figure 4**), tumor metastases were not found in the lymph nodes. The patient showed no signs of recurrence at the 2-year follow-up.

See Annex 1 for the timeline for the cases.

## 3 Discussion

GCT, often confused with myogenic tumors with a granulosa cell variation (16), is also known as myoblastoma or Abrikossoff tumor, as it was first described by Abrikossoff in 1926 (17). Although several case studies of GCT have been reported, it remains challenging to clinically distinguish it from malignant lesions. We presented two cases of GCT in different tissues and described the elastographic features and contrast-enhanced ultrasonographic manifestations for the first time. We aimed to offer clinicians and radiologists a more complete perspective of GCT features, which may lay a foundation for subsequent studies.

In view of the variability and clinical importance of this tumor, we briefly reviewed the conventional ultrasound features of GCT in similar studies. In reports by Patel et al., on ultrasound examination, the GCT root in the pectoral muscle presented as a hypoechoic mass with unclear boundaries (10). In addition we found that the thickness of the muscles at both ends of the mass on two-dimensional ultrasound images were asymmetric in case one. Another case report showed that a GCT located in the axillary region presented as a hypoechoic mass with unclear boundaries and posterior acoustic shadowing on gray-scale ultrasonography (9, 14). While we observed similar findings in case two of our study, the lesions in our case presented with a more twisted structure with an aspect ratio greater than one, whereas those of case six in the study by Aoyama et al. presented as an uneven mass with smooth boundaries and acoustic shadowing (18), which may have been caused by internal fibrosis (19).

Clinically and radiologically, it is challenging to differentiate these two cases from other entities. The differential diagnosis for case one included breast tumors, intramuscular benign and malignant tumors, and inflammatory lesions located in the pectoralis major muscle. The intramuscular tumors that need to be excluded included neurofibroma, hemangioma, desmoid fibroma, sarcoidosis, and fibrosarcoma (20). Inflammatory lesions in the muscular layer of the chest wall, such as proliferative myositis and tuberculosis, may also cause confusion. Concerning the radiological differential diagnosis for case two, in addition to excluding the possibility of accessory breast cancer and breast cancer in the caudate lobe of the mammary gland, various lesions involving subcutaneous soft tissue abnormalities, including inflammatory lesions (such as tuberculosis and toxoplasma infection caused by bacterial infections), metastasis of malignant tumor masses, and necrosis in axillary lymph nodes, needed to be excluded (21), as well as other benign lesions including ruptured and infected epidermoid cysts, as well as steatonecrosis. It is important to note that the final diagnosis should be based on the patient’s medical history and imaging results.

The diagnostic value of ultrasound elastography for GCT is unclear. Further studies need to be conducted to determine whether the quantitative analysis of elastography images has any diagnostic value for GCT. However, in the study by Tavare et al., the higher shear-wave velocity in deep lesions was associated with benign tumors, while the higher shear-wave velocity in subcutaneous lesions was associated with malignant tumors (22).

Contrast-enhanced ultrasonography can provide real-time data on blood perfusion to enable the qualitative and quantitative analysis of the lesions. In case one of this study, contrast-enhanced ultrasonography showed an earlier initiation of enhancement, a shorter time to peak enhancement, a more intense contrast enhancement than surrounding tissue, and an uneven high enhancement in the entire mass. This contrast-enhanced ultrasound pattern may be caused by rapid cell proliferation with resultant higher interstitial pressure, thereby causing inadequate central perfusion, and eventually, no enhancement in the necrotic area and the tumor blood vessels (23–25). In this case, the range after enhancement was slightly larger than the measurement range of gray-scale ultrasound. The size of the enhanced range may further indicate whether the tumors are invasive, which might further aid surgeons to determine and plan the surgical resection range. The dynamic enhancement curves showed a pattern of rapid washout and relatively rapid outflow suggestive of malignancy in this patient. To the best of our knowledge, perfusion-type GCT has not been described in contrast-enhanced ultrasonography. In contrast, the MRI scans for breast GCT reported by Maki et al. showed a pattern of rapid inflow and slow outflow at an appropriate rate (26). Case two in our study demonstrated mild enhancement around the mass, with no internal enhancement, which was similar to the enhancement mode of GCT of the breast described by Wang et al. (27). This enhanced pattern may suggest that the tumor is benign. However, it is worth noting that one of the important ultrasound features in this case is the appearance of dense shadowing, which may present a limitation of the role of contrast-enhanced ultrasound. Ultrasound specialists may be advised to scan from different angles to reduce the impact of sound shadowing.

Core needle biopsy is a useful diagnostic tool for diagnosing a GCT. However, a needle biopsy for a GCT at the deep surface of the mammary gland may cause damage to the pleura. In addition, a needle biopsy of the axilla may cause damage to its major vessels, especially when the operator is inexperienced. If the biopsy results suggest malignancy, a biopsy of the sentinel lymph node is necessary (19).

While a complete or extended resection may be necessary, there have been rare reports of post-resection recurrence. For cases with high Ki67 levels (≥10%), an aggressive resection, including the dissection of axillary lymph nodes, should be considered even if a histological diagnosis of atypical GCT is made (28). The patient in the first case was a young woman, and we considered it necessary to preserve the appearance and function of the breast; therefore, we performed breast reconstruction with a glandular fascia flap to reconstitute the shape of the right breast. She was very satisfied with her diagnosis and treatment. In contrast, the second patient only underwent extended tumor resection. While she was satisfied with the treatment, she complained that her upper limb movement was slightly restricted.

In conclusion, our cases are of clinical significance as they are rare cases of extramammary GCT encountered upon breast examination. We believe that their ultrasonographic, shear wave elasticity, and CEUS characteristics, along with detailed clinical information provided here will increase the awareness of clinicians, aid in avoiding misdiagnoses, and enable the formulation of an appropriate treatment plan.

## Data availability statement

The original contributions presented in the study are included in the article/**Supplementary Material**. Further inquiries can be directed to the corresponding authors.

## Ethics statement

The studies involving human participants were reviewed and approved by Bioethics Committee of West China Hospital of Sichuan University. The patients/participants provided their written informed consent to participate in this study. Written informed consent was obtained from the participant/patient(s) for the publication of this case report.

## Author contributions

MZ collected the case information and wrote the paper. YP and YC edited and reviewed the paper. HX obtained the pathological images and was responsible for the pathology content of the text. All authors contributed to the article and approved the submitted version.
