# Supplementary material for: Multimodal ultrasonography findings of extramammary granular cell tumors: Two case reports
Source: Front Oncol. 2023 Mar 20;13:1136770. doi: 10.3389/fonc.2023.1136770 (PMC10067867; doi:10.3389/fonc.2023.1136770)
Supplement: Supplementary file 1 [file Presentation_1.ppt]

## Slide 1
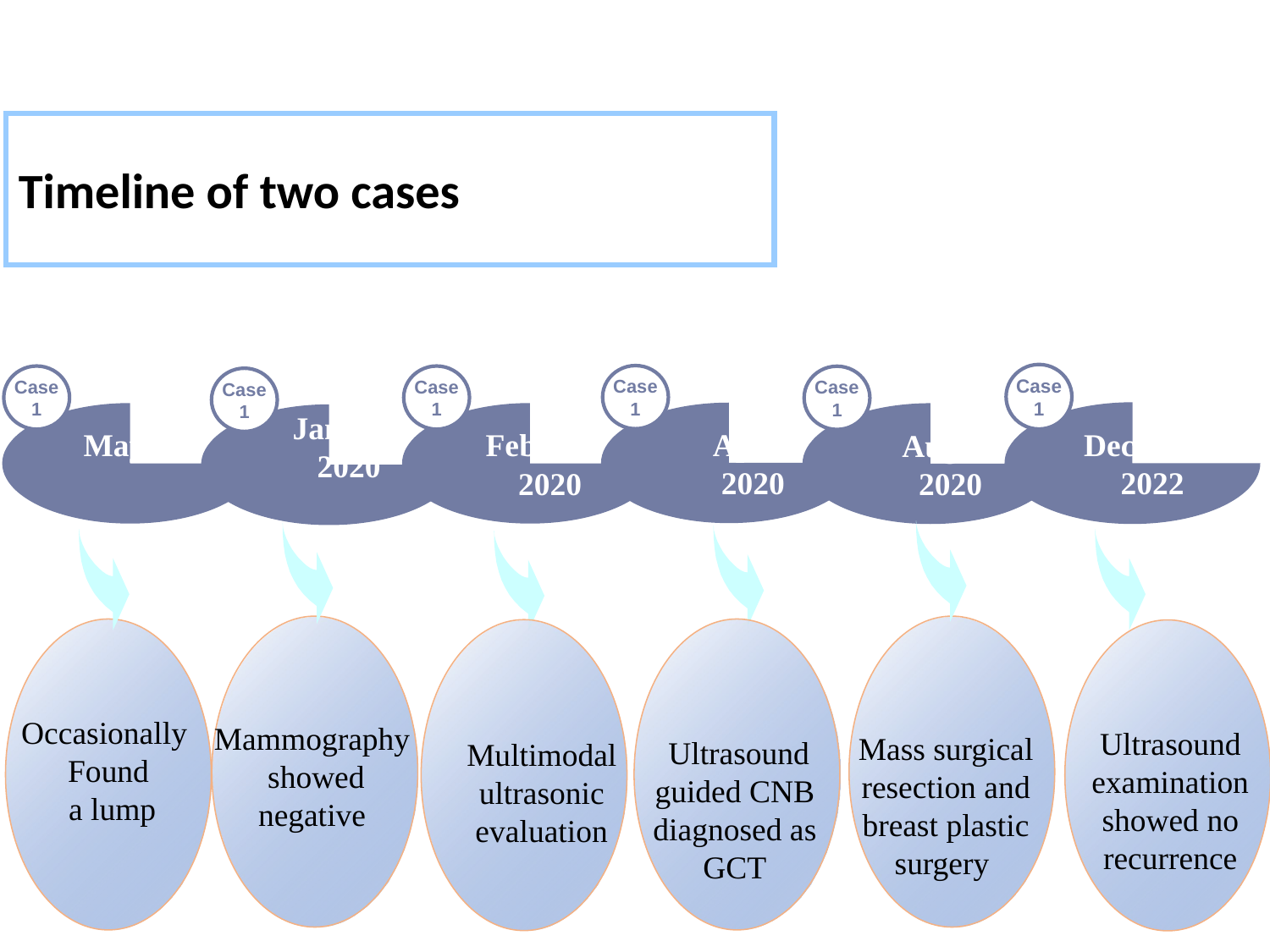

Timeline of two cases
Case 1
December 2022
Case 1
April
 2020
Case 1
May 2019
Case 1
February 2020
Case 1
August 2020
Case 1
January 2020
Occasionally
Found
 a lump
Mammography
showed negative
Ultrasound examination showed no recurrence
Mass surgical resection and
breast plastic surgery
 Ultrasound guided CNB diagnosed as GCT
Multimodal ultrasonic evaluation

## Slide 2
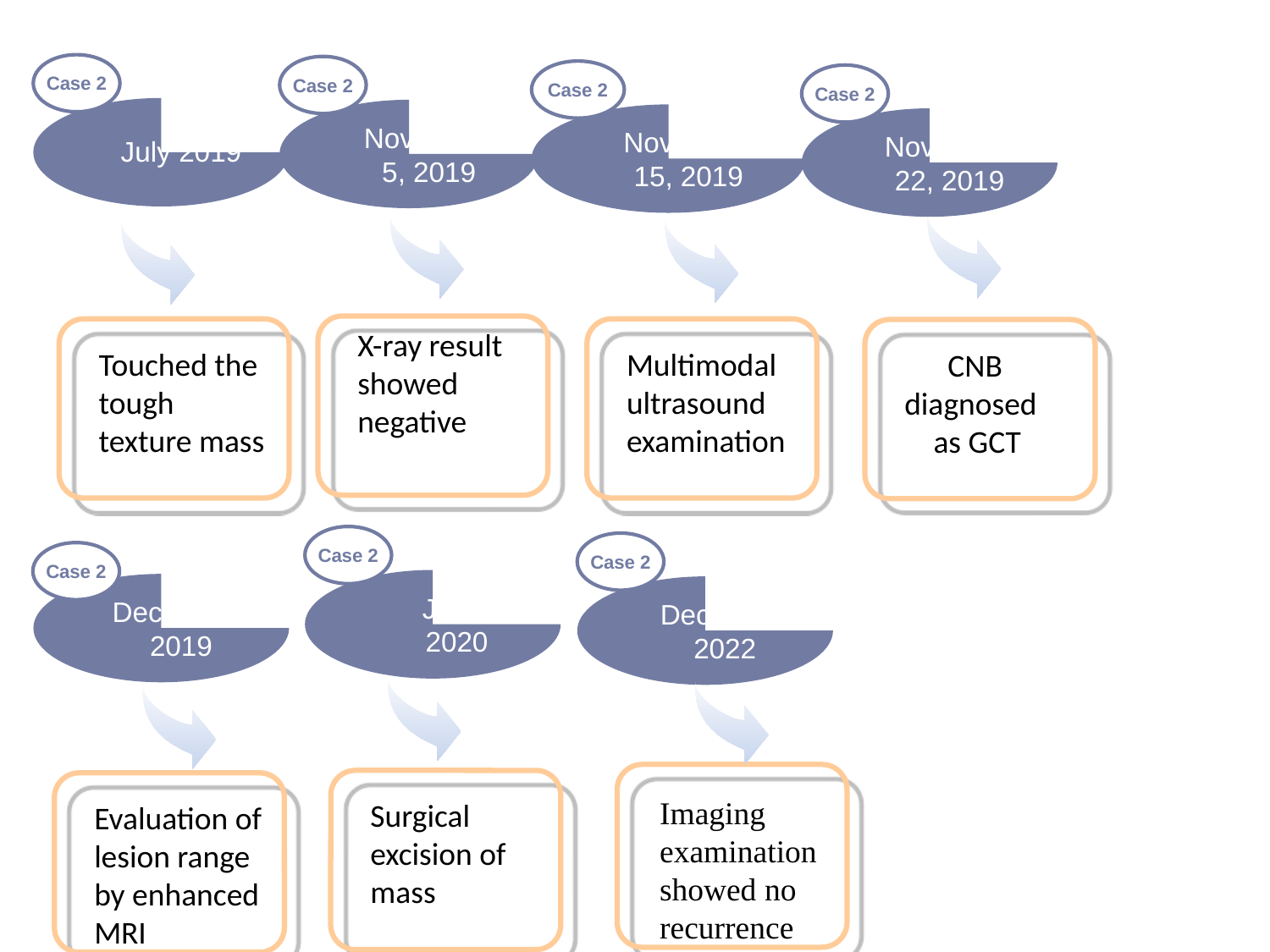

Case 2
July 2019
Case 2
November 5, 2019
Case 2
November 15, 2019
Case 2
November 22, 2019
X-ray result showed negative
Touched the tough texture mass
Multimodal ultrasound examination
 CNB diagnosed
 as GCT
Case 2
June
 2020
Case 2
December 2022
Case 2
December 2019
Imaging examination showed no recurrence
Surgical excision of mass
Evaluation of lesion range by enhanced MRI
